# Reliability and validity of different ankle MRI scanning planes for the anterior talofibular ligament injury diagnosis: a cadaveric study

**DOI:** 10.1186/s13018-019-1102-4

**Published:** 2019-02-28

**Authors:** Shengxuan Cao, Chen Wang, Xin Ma, Xu Wang, Jiazhang Huang, Chao Zhang, Kan Wang

**Affiliations:** 1 Department of Orthopedics, Huashan Hospital, Fudan University, No.12, Middle Wulumuqi Road, Jingan District, Shanghai, 200040 China; 2 Department of Radiology, Huashan Hospital, Fudan University, No.12, Middle Wulumuqi Road, Jingan District, Shanghai, 200040 China

**Keywords:** Anterior talofibular ligament, MRI, Scanning planes, Diagnostic accuracy, Cadaveric study

## Abstract

**Background:**

The objective of the current study is to compare reliability, accuracy, sensitivity, and specificity in magnetic resonance imaging (MRI) evaluation of anterior talofibular ligament (ATFL) among the routine axial scanning plane, oblique axial-coronal scanning plane, and oblique axial-sagittal scanning plane.

**Methods:**

Twenty cadaveric feet were studied. ATFL was exposed before scanning. Routine axial, oblique axial-coronal, and oblique axial-sagittal MRI scanning of 20 ATFL-intact cadaveric feet were acquired utilizing a 1.5-T MRI unit. The scans were repeated on the 20 cadaveric feet after the ATFL was artificially injured. In total, 120 sets of MR images were obtained and were randomly numbered. Three independent observers who were blinded to the experiment evaluated the images. Interobserver agreement, sensitivity, specificity, and accuracy were calculated and compared between different scanning planes utilizing the McNemar test.

**Results:**

The interobserver agreement was fair to good (kappa, 0.55 to 0.65) in the routine axial plane, fair to good (kappa, 0.557 to 0.75) in the oblique axial-sagittal plane, and excellent (kappa, 0.85 to 0.95) in the oblique axial-coronal plane. The accuracy was significantly higher when utilizing oblique axial-coronal MRI scanning than routine axial MRI scanning (reader 1: *p* = .018; reader 2: *p* = .005).

**Conclusions:**

The diagnostic accuracy of oblique axial-coronal plane MRI was higher than the routine axial plane concerning ATFL injury, and the interobserver agreement was excellent. The oblique axial-coronal plane could be added to the MRI scanning protocol during clinical practices to improve the diagnostic accuracy of ATFL injury.

## Introduction

An ankle sprain is a common injury in sports and daily living. After an ankle sprain, anterior talofibular ligament (ATFL) is the most frequently injured ligament. If injured ATFL is untreated or treated inappropriately, some patients will develop residual symptoms like pain, giving way, feeling of instability, or early-onset osteoarthritis [1]. Some cohort studies estimate more than 20% ankle sprain patients eventually developed persistent symptoms [2]. The patients’ history and clinical manual tests are important for the diagnosis of acute or chronic ATFL injury. However, a systemic review investigated the accuracy of clinical manual tests and suggested that ligament injury cannot be ruled out even when clinical manual tests are negative [3]. When the results of clinical tests are ambiguous, imaging techniques like magnetic resonance imaging (MRI), stress radiography, and ultrasonography could be helpful. MRI is widely utilized in diagnosing ankle ligament injury [4, 5] and could also provide information about concomitant lesions and influence the precise operative technique for a certain patient [6].

ATFL courses anteriorly, medially, and inferiorly from fibula to talus and sometimes cannot be delineated clearly on a routine axial plane in MRI [7]. To better delineate ATFL in ankle MRI, two approaches were utilized. One is to reposition the tested foot with tape or custom-made device [7, 8]. The other is to utilize oblique axial plane scanning or multiplanar reconstruction of MR images, parallel to ATFL course [9, 10]. The second method was more convenient in clinical practice. ATFL was reported to be directed at approximately 45° medially from the sagittal plane and at approximately 25° inferiorly from the horizontal plane [11, 12]. The oblique axial-coronal plane and oblique axial-sagittal plane were parallel to ATFL course in different planes. Kim et al. reported the oblique axial-coronal plane (ATF view) from the center of talus to the center of navicular delineated full-length view of ATFL better than routine axial view [13]. Previous studies reported grades of injury, subjective scales of optimal visualization and the rates of full-length view in an oblique axial plane compared with the routine axial plane. However, reliability and diagnostic accuracy of the oblique axial plane have not been investigated. This limited the clinical application of oblique MRI scanning planes. The objective of the current study is to compare reliability, accuracy, sensitivity, and specificity in MRI evaluation of cadaveric ATFL among routine axial plane, oblique axial-coronal plane, and oblique axial-sagittal plane. Our hypothesis is that both oblique axial-coronal MRI scanning and oblique axial-sagittal MRI scanning will deliver better reliability and diagnostic performance compared to routine axial MRI scanning.

## Methods and materials

### Specimens

This study was approved by the Institutional Review Board of our institution. Twenty-two fresh below-knee cadaveric feet were first included in the current study. Specimens with evidence of previous operative procedures or trauma were not included. Specimens were kept frozen at − 18 °C in a freezer at the Department of Anatomy of our institution and were thawed at room temperature for 24 h before the experiment.

Through a 4-cm incision starting from the anterior border of the distal fibula to subtalar joint, after fat and connective tissue were removed, the ATFL of each cadaveric foot was exposed. The ATFLs of two cadaveric feet were found to be discontinuous and were excluded. The ATFLs of the rest 20 cadaveric feet were confirmed to be intact and tight. These 20 cadaveric feet were subsequently studied.

### MRI

**Fig. 1:**
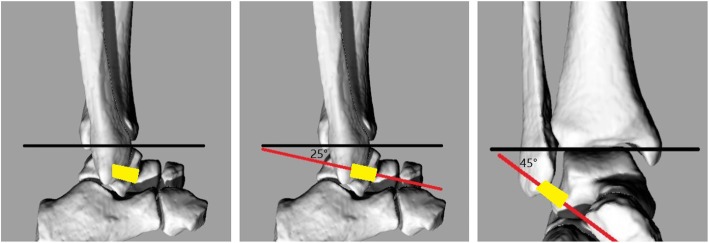
Schematic diagrams of routine axial plane, oblique axial-coronal plane, and oblique axial-sagittal plane MRI scanning. The black line indicates the tibiotalar joint line. The red line indicates different scanning planes. The yellow quadrilateral indicates ATFL. The oblique axial-coronal plane was at 25° with the tibiotalar joint line, and the oblique axial-sagittal plane was at 45° with the tibiotalar joint line

After the exposure of ATFL, the cadaveric feet were imaged with ankles taped in approximate neutral position. MR images were acquired using a 1.5-T unit (Signa Excite HD, GE Healthcare, Milwaukee, USA) and standard ankle coil. Fast spin-echo T1-weighted and T2-weighted images were obtained in the routine axial plane, oblique axial-coronal plane, and oblique axial-sagittal plane. MRI parameters were listed as follows: repetition time/echo time, 400/12 ms for T1-weighted and 3600/85 ms for T2-weighted images; echo train length, 3 for T1-weighted and 20 for T2-weighted images; field of view, 180 mm; slice thickness, 3 mm; and interslice gap, 1.5 mm. The routine axial plane, oblique axial-coronal plane, and oblique axial-sagittal plane were obtained as the schematic diagram depicted (Fig. 1). The routine axial plane was obtained parallel to the tibiotalar joint line. The oblique axial-coronal plane was obtained at an angle of 25° with the tibiotalar joint line according to anatomic studies [7, 12], while the oblique axial-sagittal plane was obtained at an angle of 45° with the tibiotalar joint line [7, 11, 12]. Sixty sets of MR images were acquired for 20 intact specimens.

**Fig. 2:**
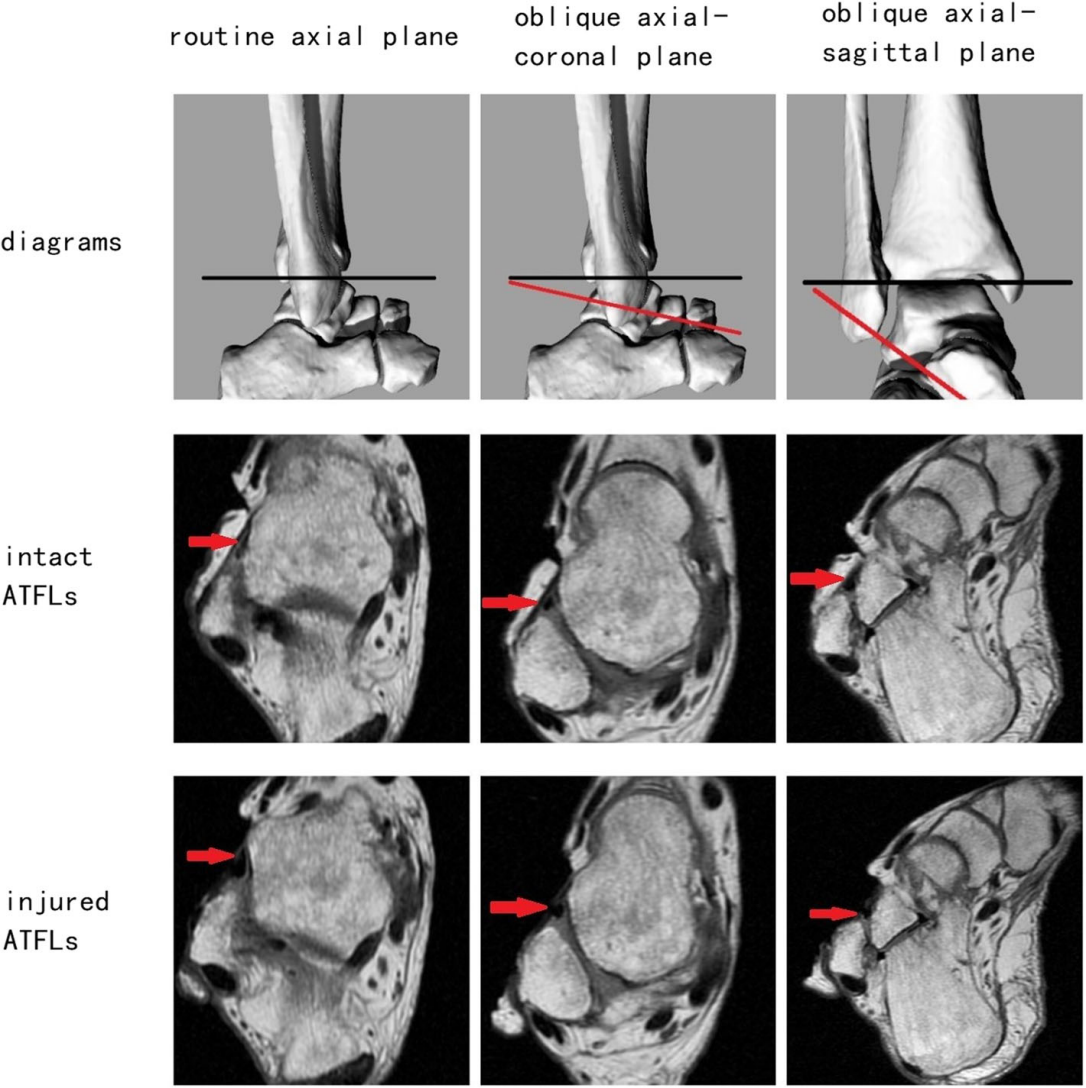
One below-knee cadaveric right foot with intact and artificially injured ATFLs utilizing different scanning planes. Red arrow indicates ATFL

After the MRI of intact ATFL was obtained, the ATFL was cut in its middle part. The routine axial plane, oblique axial-coronal plane, and oblique axial-sagittal plane images of injured ATFL were obtained with the same parameters as previously described (Fig. 2). One MRI scan contained both T1-weighted and T2-weighted images. Therefore, six MRI scans were obtained for each specimen in this experiment, and in total, 120 sets of MR images were acquired.

### Image review and statistical analysis

The 120 sets of MR images were randomly numbered. ATFL was diagnosed as injured if discontinuity presented in at least one slice of MRI scanning. Three observers (readers 1, 2, and 3), who were blinded to the experiment design and order of the images, reviewed all these images and decided the integrity of ATFL independently.

Statistical analyses were performed using SPSS 19.0. The accuracy, sensitivity, and specificity of the routine axial plane, oblique axial-coronal plane, and oblique axial-sagittal plane MRI were calculated respectively. The McNemar test was utilized to compare sensitivity, specificity, and accuracy between the different MRI scanning approaches. Interobserver agreements were assessed utilizing the kappa tests. The results of kappa tests were interpreted as follows: a kappa value of 0.81 to 1 indicated excellent correspondence, a kappa value of 0.61 to 0.8 indicated good correspondence, a kappa value of 0.41 to 0.6 indicated fair correspondence, and a kappa value below 0.4 indicated poor correspondence [14].

## Results

The interobserver agreement of three scanning methods was listed in Table 1. The interobserver agreement regarding the injury of ATFL was fair to good (kappa, 0.55 to 0.65) in routine axial planes, fair to good (kappa, 0.557 to 0.75) in oblique axial-sagittal planes, and excellent (kappa, 0.85 to 0.95) in oblique axial-coronal planes.

**Table 1:** Interobserver agreement between readers

	Reader 1 versus 2	Reader 1 versus 3	Reader 2 versus 3
Routine axial plane	0.65	0.60	0.55
Oblique axial-coronal plane	0.90	0.85	0.95
Oblique axial-sagittal plane	0.75	0.557	0.70

The diagnostic performance of each scanning plane was listed in Table 2. For reader 1, the sensitivities of routine axial, oblique axial-coronal, and oblique axial-sagittal MRI scanning were 0.65, 0.85, and 0.6 respectively. The specificities of routine axial, oblique axial-coronal, and oblique axial-sagittal MRI scanning were 0.65, 0.9, and 0.75 respectively. The accuracy of reader 1 was significantly higher (*p* = .018) when utilizing oblique axial-coronal MRI scanning (accuracy = 0.875) than routine axial MRI scanning (accuracy = 0.65).

**Table 2:** Sensitivity, specificity, and accuracy of different scanning planes

	Routine axial plane			Oblique axial-coronal plane			Oblique axial-sagittal plane		
	Reader 1	Reader 2	Reader 3	Reader 1	Reader 2	Reader 3	Reader 1	Reader 2	Reader 3
Sensitivity	0.65 (13/20)	0.6 (12/20)	0.85 (17/20)	0.85 (17/20)	0.95 (19/20)*	0.95 (19/20)	0.6 (12/20)	0.75 (15/20)	0.75 (15/20)
Specificity	0.65 (13/20)	0.75 (15/20)	0.85 (17/20)	0.9 (18/20)	0.9 (18/20)	0.95 (19/20)	0.75 (15/20)	0.75 (15/20)	0.85 (17/20)
Accuracy	0.65 (26/40)	0.675 (27/40)	0.85 (34/40)	0.875 (35/40)*	0.925 (37/40)*	0.95 (38/40)	0.675 (27/40)	0.75 (30/40)	0.8 (32/40)

*p < .05 compared to the routine axial plane of the same reader

For reader 2, the sensitivity of reader 2 was also significantly higher (*p* = .008) when utilizing oblique axial-coronal MRI scanning (sensitivity = 0.95) than routine axial MRI scanning (sensitivity = 0.6). The specificities of routine axial, oblique axial-coronal, and oblique axial-sagittal MRI scanning were 0.75, 0.9, and 0.75 respectively. The accuracy of reader 2 was also significantly higher (*p* = .005) when utilizing oblique axial-coronal MRI scanning (accuracy = 0.925) than routine axial MRI scanning (accuracy = 0.675).

For reader 3, the sensitivities of routine axial, oblique axial-coronal, and oblique axial-sagittal MRI scanning were 0.85, 0.95, and 0.75 respectively. The specificities of routine axial, oblique axial-coronal, and oblique axial-sagittal MRI scanning were 0.85, 0.95, and 0.85 respectively. The accuracies of routine axial, oblique axial-coronal, and oblique axial-sagittal MRI scanning were 0.85, 0.95, and 0.8 respectively.

## Discussion

The oblique axial plane, which is parallel to the course of ATFL, was reported to delineate ATFL more clearly in MRI scanning [13]. However, the application of oblique scanning planes is uncommon in clinical practice, because the reliability and validity of the oblique axial plane were still unknown. The current cadaveric study investigated the interobserver agreement and diagnostic performance of two kinds of oblique axial plane. According to our results, the diagnostic performance of the routine axial plane and oblique axial-sagittal plane were comparable. The diagnostic accuracy of the oblique axial-coronal plane was higher than the routine axial plane.

MRI is helpful when the diagnosis of ATFL injury is uncertain after careful inquiry of the patient’s history and physical tests. MRI is also frequently performed to confirm or exclude the presence of concomitant lesions of ligamentous injury and influence the precise operative technique for a certain patient. Ultrasonography and stress radiograph were also utilized in the diagnosis of ATFL injury. However, ultrasonography is of limited value in assessing bone or cartilage lesions and may be much less accurate in less experienced hands. Stress radiograph was reported to have a high rate of false negative results [15].

The previously reported diagnostic efficacy of MRI was listed in Table 3. Due to variable MRI parameters, gold standards, and injury types in individual study, the diagnostic efficacy of MRI varied largely. The sensitivity, specificity, and accuracy of MRI diagnosing ATFL injury were reported to be 0.5 to 1, 0.5 to 1, and 0.588 to 1 respectively, and the interobserver agreement was reported to be 0.4 to 0.939 (kappa value) [4–6, 10, 16–27]. The sensitivity, specificity, and accuracy of routine axial MRI scanning diagnosing ATFL injury in the current study were 0.65 to 0.85, 0.65 to 0.85, and 0.65 to 0.85. These results were comparable with other studies.

**Table 3:** Diagnostic accuracy of MRI in previous studies

Author	Publication year	Number of subjects	Injury type	Gold standard	MRI parameters	Sensitivity	Specificity	Accuracy	Reliability
Jolman	2017	187	Chronic	Patients: operative findings; control: clinical findings	1.5-T or 3-T MRI	0.826	0.533	0.71	N/A
Kim	2015	79	Chronic	Arthroscopic findings	1.5-T MRI	0.764 to 0.836	0.833 to 0.917	0.785 to 0.861	0.915 (intraclass correlation coefficient)
Morvan	2018	22	Chronic	Arthroscopic findings	1.5-T MRI with T2-weighted sequence	0.857 to 0.875	0.867 to 0.929	0.864 to 0.909	0.55 to 0.87 (kappa)
Tan	2016	82	Chronic and acute	Operative findings	3-T MRI	0.64 to 0.78	0.86 to 0.80	0.74 to 0.79	N/A
Yi	2016	35	Not mentioned	Arthroscopic findings	3-T MRI with 3D T2-weighted FSE and 2D T2-weighted FSE sequences	N/A	N/A	N/A	0.533 to 0.695 (kappa)
Park	2016	101	Not mentioned	Operative or clinical findings	3-T MRI with 2D T2-weighted FSE and 3D VISTA sequences	0.935 to 1	0.843 to 0.957	0.891 to 0.96	0.887 to 0.939 (kappa)
Park	2012	48	Chronic	Operative findings	1.5-T MRI with T1-weighted, spin-echo, proton density-weighted, and T2-weighted FSE sequences	0.75	0.86	0.875	N/A
Joshy	2010	24	Chronic	Arthroscopic findings	1-T MRI with T1-weighted, T2-weighted, and proton density-weighted sequences	0.67	1	0.917	N/A
Oae	2010	34	Not mentioned	Arthroscopic findings	1.5-T MRI with T2-weighted sequence	0.92	1	0.97	N/A
Kreitner	1999	18	Acute	Operative findings	1.5-T MRI	1	N/A	1	N/A
Breitenseher	1997	60	Acute	Operative or clinical findings	Not mentioned	0.74	1	N/A	N/A
Gaebler	1997	25	Acute	Operative findings	1-T MRI with T1-weighted, T2-weighted weighted sequences	1	N/A	1	0.4 to 0.65 (kappa)
Chandnani	1994	17	Chronic	Operative findings	1.5-T MRI with T1-weighted and T2-weighted sequences	0.5	1	0.824	N/A
Kumar	2007	58	Chronic	Operative findings	1.5-T MRI	0.87	0.6	0.724	N/A
Verhaven	1991	17	Acute	Operative findings	1.5-T MRI with 3D FISP sequence	1	0.5	0.944	N/A
Lee	2011	34	Chronic	Arthroscopic findings	3-T MRI with T2-weighted sequence	0.606 to 0.969	N/A	0.588 to 0.941	0.48 to 0.93 (kappa)

*N/A* not available

To better delineate a ligament, the MRI plane needs to be parallel to the long axis of the ligament. Previous anatomic study of ATFL delineates the course of ATFL from fibula to talus. The fibular origin of ATFL was reported to be 10 to 13.8 mm proximal to the tip of the fibula [28], which is approximately the midpoint between the inferior tip and anterior tubercle of the fibula. The talar insertion of ATFL was reported to be 14.2 to 18.1 mm to the subtalar joint or 11.3 to 14.8 mm to the anterolateral corner of the talar body [28], which is approximately the midpoint between the lateral talar process and the anterolateral corner of the trochlea. ATFL was reported to be directed at approximately 45° medially from the sagittal plane and at approximately 25° inferiorly from the horizontal plane [11, 12]. The oblique axial-coronal plane and oblique axial-sagittal plane utilized in the current study were parallel to the ATFL course in different planes.

The oblique axial-coronal plane was also referred to as the ATF view in previous studies [13]. Kim et al. reported the full length of 97.4% ATFL was delineated utilizing the ATF view [13]. In the current study, the accuracy of the oblique axial-coronal plane was significantly higher compared to the routine axial plane for readers 1 and 2 (reader 1: *p* = .018; reader 2: *p* = .005). The accuracy for reader 3 was also raised utilizing the oblique axial-coronal plane but with the numbers available, no significant difference could be detected. The sensitivity and specificity were also raised utilizing the oblique axial-coronal plane, but only sensitivity for reader 2 showed statistical significance (*p* = .008). The interobserver agreement of the oblique axial-coronal plane was excellent. In contrast, the interobserver agreement of the routine axial plane was fair to good.

Schneck et al. [7] reported ATFL has a slightly descending course and was best delineated in 10 to 20° dorsiflexion of the tested foot. This angle is slightly less than 25° oblique axial-coronal plane utilized in the current studies. The methods of repositioning the tested foot were also reported to be effective by Farooki et al. [8] However, the optimal position of the foot was not identical among studies [7, 8]. The discrepancy in the optimal angle might be due to anatomic variance. Compared to the methods of repositioning the tested foot and multiplanar reconstruction of images, oblique plane MRI scanning was more convenient in clinical practice. It only took a couple of minutes for the additional oblique plane MRI scanning of one specimen in the current study.

The oblique axial-sagittal plane was firstly proposed in the current study. The oblique axial-sagittal plane was parallel to the course of ATFL in the coronal plane. However, this method did not show enough improvement in reliability or validity from the routine axial plane. This might be due to the large variance for angles in the coronal plane. Moreover, ATFL was a flat quadrilateral ligament [28]. In planes 45° degree from the sagittal plane, ATFL might demonstrate the full length on fewer slices than 25° oblique planes.

The oblique axial-coronal plane could be added to routine MRI scanning protocol for better diagnosis of ATFL injury. However, the routine axial plane is still irreplaceable in assessing lesions of other structures. Kim et al. [13] reported anterior inferior tibiofibular ligament injury, posterior inferior tibiofibular ligament injury, and posterior tibialis tendinitis were better delineated on the routine axial plane compared to ATF view. Park et al. [29] reported better delineation of the calcaneofibular ligament on an oblique coronal plane.

One of the limitations of the current study is that we utilized cadaveric feet rather than clinical patients to investigate the diagnostic efficacy. Tear of ATFL in vivo is more irregular, including partial tear and tear with an avulsion fracture of fibular. We simulate the tear of ATFL by a cut in its middle part. This is the most common situation during the surgery. Exposing cadaveric ligament (ATFL) by surgically removing fat and connective tissue creates the condition not quite comparable to clinical cases. Removal of fat could be a clue that surgery was performed. We also did sham operations in the intact groups to decrease the influence of exposing the ligament. The MRI signal intensities of cadaveric ATFLs were not identical as the ATFLs in a physiological environment. We were not able to assess the conditions of ATFLs through the altered MRI signal intensities and through the contrast of synovial fluid. T2-weighted images were more common in clinical practice, because they delineate the ligaments better through the contrast of synovial fluid. However, in the current cadaveric study, T1-weighted images delineate the ligaments well (Fig. 2). Compared to acute ligament injury, the proper diagnosis of chronic ligament injury is more difficult. But it is difficult to study the MRI diagnosis of chronic ligament injury in the current cadaveric study.

However, we utilized cadaveric feet in the current study for two reasons. One is that the anatomic findings can be used as a gold standard when investigating diagnostic accuracy. This was more reliable than clinical diagnosis or arthroscopic findings as other studies mentioned. Some studies utilizing clinical findings as gold standards might have difficulties in determining whether ATFLs of “healthy volunteers” were intact or not, because they could only assess “healthy volunteers” clinically rather than through direct observation of the ligament. The other reason is that the tested feet in the intact group and injured group could be strictly matched. We scanned 20 intact feet and then scanned 20 identical artificially injured feet. The images were randomly numbered, and the orders were blind to the observers. Previous studies utilizing arthroscopic findings as gold standards might have difficulties in collecting adequate true negative patients, because most patients who underwent arthroscopic procedure were injured. This situation may cast bias on the results.

Another limitation is that this experiment included only 20 specimens. The number of specimens was relatively small. However, significantly higher accuracies were still observed for oblique axial-coronal MRI scanning (reader 1: *p* = .018; reader 2: *p* = .005), and the results of the three observers showed fair to excellent interobserver agreement.

Moreover, MRI is time-consuming and expensive. So, it is difficult to send all patients for MRI. Ultrasonography and stress radiograph were also utilized in the diagnosis of ATFL injury. Studies comparing diagnosing efficacy of different MRI scanning planes with ultrasonography and stress radiograph are warranted. However, MRI is irreplaceable when clinicians need to confirm or exclude the presence of concomitant osteochondral lesions with ligamentous injury. According to our results, the oblique axial-coronal plane could be added to the MRI scanning protocol during clinical practices to improve the diagnostic accuracy of ATFL injury.

## Conclusions

The diagnostic accuracy of the oblique axial-coronal plane MRI was higher than the routine axial plane concerning ATFL injury, and the interobserver agreement was excellent. The oblique axial-coronal plane could be added to the MRI scanning protocol during clinical practices to improve the diagnostic accuracy of ATFL injury.

## Abbreviations

ATFL: Anterior talofibular ligament
MRI: Magnetic resonance imaging
